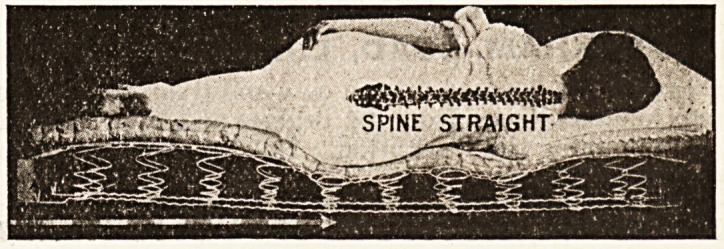# A Scientific Mattress

**Published:** 1914-09-26

**Authors:** 


### Institutional Needs.

### A SCIENTIFIC MATTRESS.

A mattress which cannot sag, which moulds itself to the figure, and which always keeps the spine straight and the abdomen undistorted, is manifestly of importance not only in conditions of disease, but as a precaution if health, proper rest, and carriage are to be preserved and maintained. The accompanying illustrations show more clearly than any description how effectually this result is arrived at by the "Staple's" patent mattress. The results are interesting as showing the practical possibilities of the spiral spring, which is the individual feature of the "Staple's" mattress. These springs yield naturally at those points of the figure where pressure is greatest and the curves most marked, and the powerful frame in which the springs are set effectually prevents the sag which is the usual accompaniment of regular wear on a woven-wire mattress. The result of the principle here applied is that the spine can never rest in any other than the straight position, while at the same time the mattress "fits" the body at every point. The prices vary according to size, from 39s. (2 ft. 6 in.) to 48s. (5 ft.), and can be made to fold at an extra cost of 3s.

## Figures and Tables

**Figure f1:**
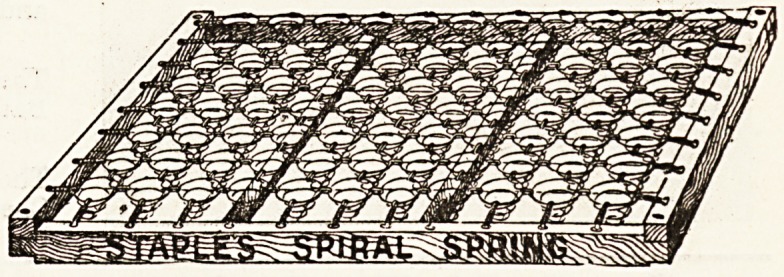


**Figure f2:**
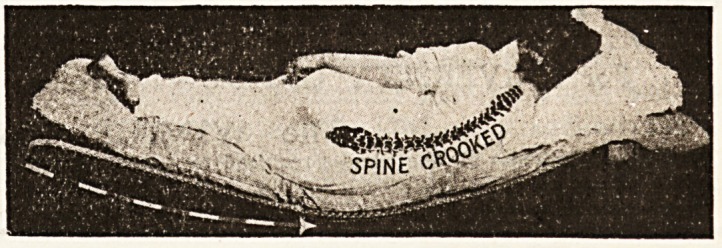


**Figure f3:**